# The Cuban Murder of Medical Students

**Published:** 1871-12

**Authors:** 


### Editorial Department.

### The Cuban Murder of Medical Students.

The history of crime and infamy does not furnish a more fearful story than the recent account of the murder of the student boys in the Medical College in Havana. A thrill of indignation moves every human heart at its recital, and language is incapable of expressing the horror of such inhumanity. We copy the following newspaper account of the tragedy, believing that such barbarity should stand recorded as history of a race of Fiends and Devils.

" Again our duty to the public compels us to record the horrible butchery of eight beardless boys by the military assassins, " volunteers," at Havana.

The heart grows faint as the eye scans the particulars of the many flagrant outrages perpetrated on the inoffensive, upon the plea of military necessity. It seems that, on Thursday, November 23d, a party of medical students visited an old cemetery, long since given up as a burial-place, and while strolling about, were detected by one of the volunteer soldiers—from the like of whom the Lord delivered any people—who made some insulting remarks to the boys which they retorted. The volunteer staid some time, and passing by the niche in which the body of Castanon—whom the Spaniards venerate as a political martyr, and who was killed by the Cubans in Key West—is deposited, noticed that the glass which covers the end of the niche was marked, apparently with a diamond, and that some letters uncomplimentary to Castanon had been scribbled on it. The volunteer called the attention of the curate to the fact, and rushed out, returned with several companions. This was the whole offence that had been committed. The boys had not been near the niche of Castanon, but as they happened to frequent the cemetary, using it partly as a playground, and partly for the collection of human bones for the study of osteology, they were accused and arrested. On Monday a court martial was convened, composed, first, of all the officers of the regular army; but as they refused to find cause for trial, another was subsequently assembled, which belonged body and soul to the volunteers. The poor victims were drafted or chosen by lot, one out of every five. The oldest of the eight was not eighteen years of age, and one was not fourteen; and although all communication with any of the forty-two has been impossible since, it has been proved that of the eight executed, three at least had not been in the cemetery. The thirty-one not sentenced to death by lot were condemned to the chain-gang for periods varying from two to ten years; and the sentences of these latter were carried into such immediate execution, that they appeared with their hair cut short, in convicts' dress, and guarded by armed volunteers, at the murdering of their classmates, which they were forced to witness.

At four o'clock in the afternoon, a detachment of volunteers was seen to issue from the jail, closely followed by several priests, by the students sentenced to death, and by the commander of the place, Colonel Villalonga. Not a sound was heard, the young men thus cruelly sentenced to meet an early and sudden death marched bravely forward. They knelt down, muttering a prayer, the firing party of the volunteers were drawn up in order, the command to make ready was almost whispered, the commanding officer of the volunteers turning his head aside to hide his emotion. Then came another short command to take aim, immediately followed by the fatal word " fire," when all were seen lying on the ground, four motionless, and four in the last agonies, from which kindly bullets relieved them.

Such are the details of the murder. When we read that the assassins marched through the city—a mob feared by all local authorities—shouting, " Death to the students!" and demanding the right to discharge their rifles at pleasure; when we know that many students, arrayed in the obnoxious costume of the chain-gang, are now sweeping the streets of Havana, by the decree of the model court-martial; when we are assured that the bodies of the youthful victims were denied their relatives for Christion burial; and when, as a consequence of the murders, fathers, mothers, sisters, and relatives are shrieking throughout the city—hopeless cases of insanity—every sympathetic feeling is wrought to the highest pitch of indignation, and an appalled civilization joins in the cry: "Good God! how long?" At this rate, the voluuteers of Havana will far surpass the Communists of Paris in atrocity.

